# The protective role of commensal gut microbes and their metabolites against bacterial pathogens

**DOI:** 10.1080/19490976.2024.2356275

**Published:** 2024-05-26

**Authors:** Liqin Cheng, Mário S. P. Correia, Shawn M. Higdon, Fabricio Romero Garcia, Ioanna Tsiara, Enrique Joffré, Åsa Sjöling, Fredrik Boulund, Elisabeth Lissa Norin, Lars Engstrand, Daniel Globisch, Juan Du

**Affiliations:** a Centre for Translational Microbiome Research (CTMR), Department of Microbiology, Tumor and Cell Biology, Stockholm, Sweden; b The Department of Pathophysiology, School of Basic Medicine Science, Central South University, Changsha, China; c Department of Chemistry - BMC, Science for Life Laboratory, Uppsala University, Uppsala, Sweden; d Department of Chemistry and Molecular Biology, University of Gothenburg, Göteborg, Sweden; e Science for Life Laboratory, Stockholm, Sweden

**Keywords:** Commensal bacteria, Salmonella, metabolite, adenosine, adenine, antibiotic-resistant, dipeptides

## Abstract

Multidrug-resistant microorganisms have become a major public health concern around the world. The gut microbiome is a gold mine for bioactive compounds that protect the human body from pathogens. We used a multi-omics approach that integrated whole-genome sequencing (WGS) of 74 commensal gut microbiome isolates with metabolome analysis to discover their metabolic interaction with *Salmonella* and other antibiotic-resistant pathogens. We evaluated differences in the functional potential of these selected isolates based on WGS annotation profiles. Furthermore, the top altered metabolites in co-culture supernatants of selected commensal gut microbiome isolates were identified including a series of dipeptides and examined for their ability to prevent the growth of various antibiotic-resistant bacteria. Our results provide compelling evidence that the gut microbiome produces metabolites, including the compound class of dipeptides that can potentially be applied for anti-infection medication, especially against antibiotic-resistant pathogens. Our established pipeline for the discovery and validation of bioactive metabolites from the gut microbiome as novel candidates for multidrug-resistant infections represents a new avenue for the discovery of antimicrobial lead structures.

## Introduction

Multi-drug resistant pathogens have become a critical public health burden worldwide.^1,2^ It is estimated that 70% of hospital-acquired infections are resistant to one or more antibiotics. Antibiotics account for 80% of the medications given to women during pregnancy and are administered to 35% of pregnant women in Western countries, which brings the potential risk of vertical transmittance of antibiotic resistance from mothers to their offspring.^3,4^ Pathogens such as *Salmonella* are a common cause of diarrhea and the leading cause of diarrheal disease-associated deaths.^5^ The antimicrobial resistant ESKAPE pathogens (*Enterococcus faecium*, *Staphylococcus aureus*, *Klebsiella pneumoniae*, *Acinetobacter baumannii*, *Pseudomonas aeruginosa*, and *Enterobacter*) are reported as the major cause of life-threatening nosocomial infections throughout the world.^6,7^ In combination with improvements in antibiotic resistance measurement, the discovery of new treatment options to combat pathogens is urgently needed, especially for antibiotic-resistant bacteria. Identifying bioactive compounds from gut and environmental microbiomes that target antibiotic-resistant bacteria is an important new strategy.

Commensal bacteria constitute the normal and healthy microbiota that have co-evolved with humans and have essential roles in supplying nutrients and defending against pathogen invasion.^8,9^ Previous reports also demonstrated the strong effects of antibiotic treatment on microbial communities with a fundamental reduction in the richness and diversity of the gut microbiota.^10–12^ Notably, disturbances of the healthy microbiome such as antibiotic treatment can lead to impaired colonization resistance and an increased susceptibility to new infections.^2,13^

The gut microbiome represents a widely unexplored rich reservoir for bioactive compounds capable of protecting the human body from pathogen invasion. Of particular interest is the discovery of two types of gut microbiome targets for defense against pathogens that include i) direct interactions of commensal bacteria with pathogens and ii) metabolites produced, excreted, or converted by gut bacteria. Fecal microbiota transplants (FMTs) are an effective treatment for *Clostridioides difficile* infections and are also a promising therapy option for other gastrointestinal diseases including irritable bowel syndrome and even neurological disorders such as Parkinson’s disease.^14–16^ However, the content of fecal microbiota from donors is highly dynamic and FMT also brings a potential risk of donor-derived disease transmission.^17^ More consistent microbial consortia with a defined amount of viable bacteria were developed to treat recurrent *C. difficile* infection and inflammatory bowel diseases (IBD) in clinical trials with promising results.^18–20^ In our previous study, a large reduction of *Salmonella Enteritidis* growth was identified after the administration of anaerobically cultivated human intestinal microbiota (ACHIM).^10,21,22^

While employing bacterial strains to reduce pathogenic growth is a powerful strategy, recent studies have demonstrated that microbiome-derived metabolites such as lipids, amino acids, and short-chain fatty acids (SCFAs) have multiple effects on interfering with pathogenic invasion and host signaling processes.^23–25^ The impact of propionate, which is produced by commensal bacteria, was identified as altering *Salmonella* colonization.^26^ Furthermore, succinate was investigated as a signaling metabolite for *Salmonella* virulence.^27^ Antimicrobial peptides (AMPs) are reported to have significant efficacy against a wide spectrum of bacteria while causing little or no antimicrobial resistance and most bioactive antibiotics are derived from microorganisms as secondary metabolites.^28,29^ Multiple omics-based strategies with high-throughput screening, metagenomics, and metabolomics have also been developed for the biodiscovery of a new class of microbial natural products to counter antibiotic resistance.^30,31^

In this study, we employed a multi-omics strategy including the whole-genome sequencing (WGS) of commensal gut microbiome isolates, and state-of-the-art metabolome analysis to identify metabolic interaction of gut microbiome isolates with *Salmonella*. Our comparative genomic approach enabled the selection of a diverse subpopulation for the following functional assays. We identified the chemical structure for most of the top altered metabolites in the supernatant of *Salmonella* growth cultures compared to co-culture supernatants of the selected commensal gut microbiome isolates. The validated metabolites were further tested for their growth inhibition properties toward a group of antibiotic-resistant pathogens (*S. Enteritidis*, *S. Typhimurium*, *S. aureus*, *Escherichia coli*, and *K. pneumoniae*). Our results demonstrate a powerful example that the gut microbiome is a gold mine for bioactive products including previously unknown metabolites. Our reported strategy represents a pipeline for the discovery and *in vivo* validation of metabolites from the gut microbiome as novel potential interventions for multi-drug-resistant pathogens.

## Results

### Gut microbiota bacterial isolates exhibited substantial genomic diversity

**Figure 1.**
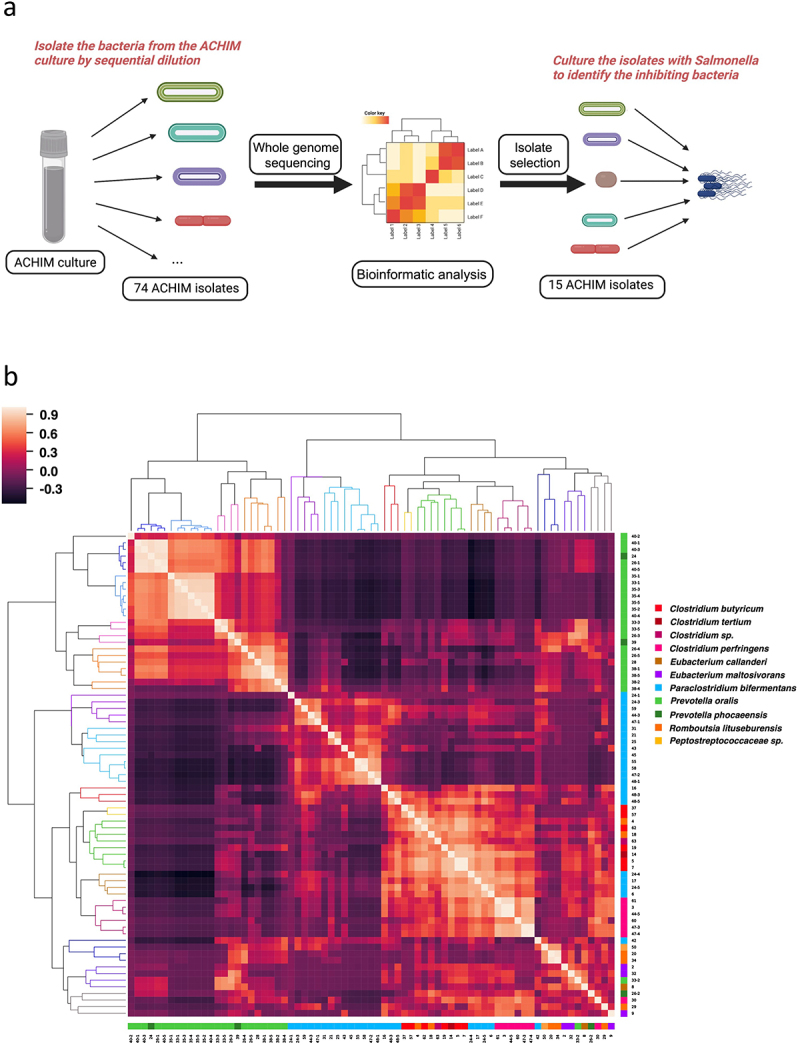
Gut microbiota isolate whole-genome sequencing (WGS) similarity and *Salmonella* co-culture experimental workflow.

Previous investigations into commensal gut microbiota cultures demonstrated their strong potential for the inhibition of *Salmonella* growth.^10^ To determine which bacterial species play key roles in *Salmonella* inhibition, we generated a collection of 112 bacterial isolates from the ACHIM cultures and subjected them to WGS analysis. A bioinformatic examination of each isolate’s WGS library revealed that some of the isolates are genomically identical. In total, 74 ACHIM isolates with distinct genomes were found and cultured (Figure 1a). Assessing the pairwise Average Nucleotide Identity (ANI) of these 74 isolates demonstrated a broad diversity among the isolated population with values ranging from 76.8% to 97.8%.Obtaining the ANI percentage values for each unique isolate pair enabled the generation of an all-by-all similarity matrix based on calculated Pearson Correlation Coefficients, which resulted in the formation of 15 primary clades after hierarchical clustering (Figure 1b). WGS-based taxonomic classification of each isolate using the Lowest Common Ancestor (LCA) algorithm revealed that most terminal nodes comprising each ANI-derived clade represented organisms of the following species: *Prevotella phocaeensis*, *P. oralis*, *Paraclostridium bifermentans*, *Clostridium perfringens*, *C. butyricum*, *Romboutsia litusburensis*, and *Eubacterium maltosivorans*. (Table S1 and Table S2).

(a) Schematic representation of the ACHIM isolates and *Salmonella* co-culture experiment workflow. We isolated 112 bacteria from the ACHIM culture by sequential dilution and obtained 74 different ACHIM isolates. Bioinformatic analysis of WGS data for these isolates enabled the selection of 15 ACHIM isolates for co-culture with *Salmonella* to identify isolates capable of inhibiting *Salmonella* growth. (b) Average nucleotide identity similarity matrix for the 74 gut microbiota isolates that passed bioinformatic filtering and contamination screens. The heatmap color scale indicates the calculated Pearson Correlation Coefficients of all pairwise average nucleotide identity (ANI) comparisons between isolate WGS assemblies. Positive values closer to 1 indicate high genome sequence similarity and negative values closer to -1 indicate lower genome sequence similarity. Isolates are hierarchically clustered based on similarity in correlation coefficient profiles.

**Figure 2.**
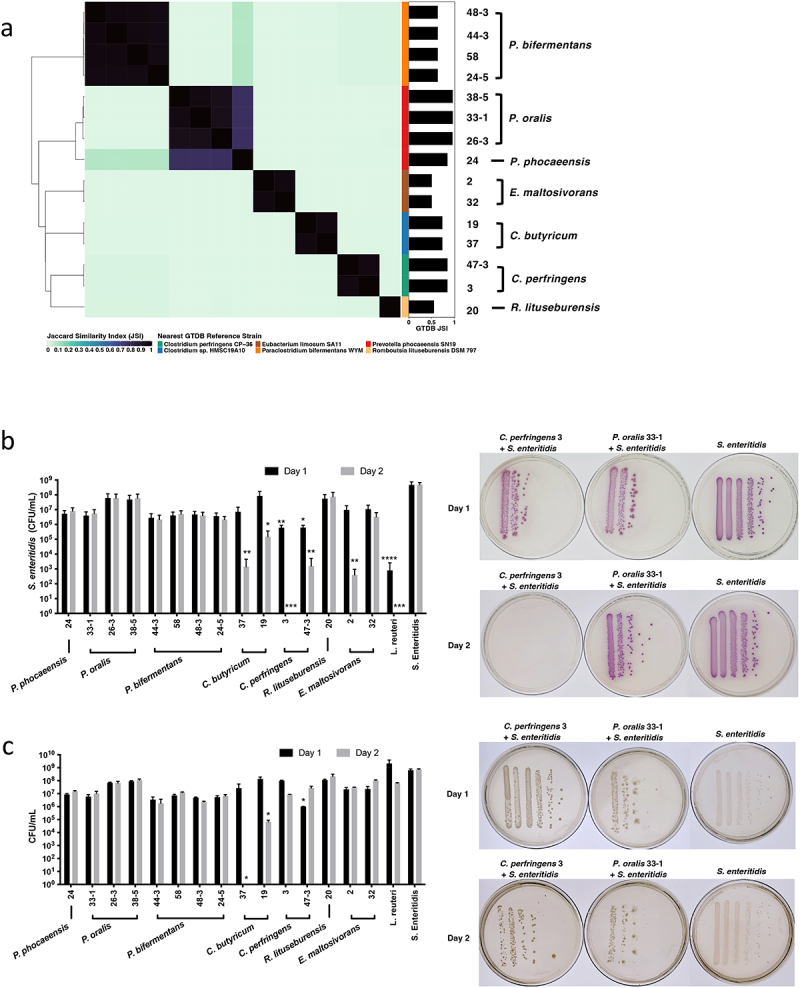
Gut microbiota isolates selectively reduce *Salmonella* growth.

We selected 15 single representative isolates of each distinguished ANI-derived clade in downstream functional assays and delved deeper into their respective WGS data. Re-examination of the respective genomic distances among the selected isolates expressed in terms of the Jaccard Similarity Index (JSI) value between two MinHash genome sketches corroborated results from the ANI analysis.^32^ However, while this analysis highlighted the genomic diversity of the selected subpopulation with JSI values ranging from zero to one, it also revealed distinct groups with substantial levels of genomic overlap (Figure 2a). Identification of a “best-match” reference organism from the Genome Taxonomy Database (GTDB) for each of the selected isolates showed the resemblance to *P. phocaeensis* SN19 (*n* = 4), *P. bifermentans* WYM (*n* = 4), *C. perfringens* CP-36 (*n* = 2), *Clostridium sp*. HMSC19A10 (*n* = 2), *Romboutsia litusburensis DSM 797* (*n* = 1), and *Eubacterium limosum* SA11 (*n* = 2) (Figure 2a and Fig. S1a).^33^

(a) MinHash genome distance and best-match WGS reference genome assignment. Heatmap values indicate the Jaccard Similarity Index (JSI) between WGS genome sketches. Values closer to 1 (darker) indicate higher similarity in nucleotide composition and values closer to 0 indicate low genome similarity. The genome taxonomy database (GTDB) bar annotations indicate the JSI between gut microbiota isolates and the most similar reference organism in the GTDB r202. (b-c) Quantification by colony forming units (CFU) of *Salmonella* growth at 24 and 48 h after co-culture. Representative images on the upper right showed the growth of *Salmonella* on *Salmonella*-selective agar plates under aerobic condition (purple color colonies). The treatment conditions were stated above the plates, and each bacterial line represented a serially diluted concentration of *Salmonella* at 24 and 48 hours after co-culture (0 to 10^5^ dilution of 10ul culture from lef to right). Representative images on the lower right showed the growth of total bacteria on YCFA agar plates under anaerobic condition (ochre color colonies). Bacteria quantifications of each co-culture mix were compared with the *Salmonella* growth in the culture medium without mixing with other bacteria by Kruskal–Wallis one-way ANOVA. Data were represented as mean ± SD in a log scale from three independent assays with triplicates. *p < .05, **p < .01, ***p < .001 and ****p < .0001.

### Gut microbiota isolates reduced *Salmonella* growth and exhibited genomic diversity

We cultured the 15 selected isolates with 10^6^ CFU of *Salmonella* for 24 and 48 h. The co-cultures of two *C. perfringens* isolates (ACHIM 3 and 47–3) manifested significant inhibition of *Salmonella* growth within 24 h (*Salmonella* selective plates after co-culture: *C. perfringens* 3: 4.27 × 10^5^ CFU/mL;*C. perfringens* 47–3: 7.60 × 10^5^ CFU/mL; Control *Salmonella*: 6.63 × 10^8^ CFU/mL) and demonstrated an even stronger inhibition on the second day (*C. perfringens* 3: 0 CFU/mL; *C. perfringens* 47–3: 2.67 × 10^3^ CFU/mL; Control *Salmonella*: 6.23 × 10^8^ CFU/mL) (Figure 2b). Two other isolates from *C. butyricum* (ACHIM 19 and 37) and one of the isolates from *E. maltosivorans* (ACHIM 2) also showed significant inhibition of *Salmonella* growth after 48 hours of co-culture (*C. butyricum* 19: 2.52 × 10^5^ CFU/mL; *C. butyricum* 37: 2.33 × 10^3^ CFU/mL; *E. maltosivorans* 2: 6.67 × 10^2^ CFU/mL; and Control *Salmonella* to 6.23 × 10^8^ CFU/mL) (Figure 2b). *Salmonella* co-cultured with isolates from *P. phocaeensis*, *P. oralis*, *P. bifermentans*, and *R. lituseburensis* did not yield significant differences compared to the control group (Figure 2b).

To follow the growth of the commensal gut microbes, we also cultured the bacteria on YCFA plates on which both gut microbes and *Salmonella* grew similarly. The growth of these total bacteria was unchanged except for *C. butyricum* 19 and *C. butyricum* 37, which had significantly decreased after 2 days in co-culture (YCFA plates after co-culture: Day 1: *C. perfringens* 3: 9.90 × 10^7^ CFU/mL; *C. perfringens* 47–3: 1.01 × 10^6^ CFU/mL; *C. butyricum* 19: 1.36 × 10^8^ CFU/mL; *C. butyricum* 37: 2.65 × 10^7^ CFU/mL; *E. maltosivorans* 2: 2.17 × 10^7^ CFU/mL; Day 2: *C. perfringens* 3: 8.70 × 10^6^ CFU/mL; *C. perfringens* 47–3: 2.69 × 10^7^ CFU/mL; *C. butyricum* 19: 6.67 × 10^4^ CFU/mL; *C. butyricum* 37: 0 CFU/mL; *E. maltosivorans* 2: 3.10 × 10^7^ CFU/mL) (Figure 2c).

Results from these *Salmonella* co-culture assays yielded two phenotypic isolate groups: a “Kill” group comprised of isolates exhibiting the strongest *Salmonella* inhibition phenotypes (*E. maltosivorans* 2, *C. perfringens* 3, and *C. butyricum* 37) and a “No-Kill” group containing isolates that equally demonstrated the weakest growth inhibition (*R. litusburensis* 20, *P. oralis* 26–3, and *P. oralis* 38–5). By comparing the colony-forming units (CFUs) on YCFA plates with those on *Salmonella*-selective plates, we observed that ACHIM, isolated from the “Kill” group, including *C. perfringens* and *E. maltosivorans*, grew after co-culture (Figure 2c). We then selected four closely related strains, according to the ANI similarity matrix (Figure 1b), to the “Kill” and “No-Kill” strains under the same cluster. For the strains close to the “Kill” group, *C. perfringens* 61 and 44–5 showed similarly significant inhibition of *S. enteritidis* growth at 24 (*C. perfringens* 61: 3.78 × 10^5^ CFU/mL isolate 61; *C. perfringens* 44–5: 3.70 × 10^5^ CFU/mL) and 48 h (*C. perfringens* 61: 2.98 × 10^5^ CFU/mL; *C. perfringens* 44–5: 3.40 × 10^5^ CFU/mL), while the viability of the isolates themselves was not affected according to the YCFA plates at 24 (*C. perfringens* 61: 5.60 × 10^7^ CFU/mL; *C. perfringens* 44–5: 4.53 × 10^7^ CFU/mL isolate 44–5) and 48 h (*C. perfringens* 61: 6.04 × 10^7^ CFU/mL; *C. perfringens* 44–5: 5.13 × 10^7^ CFU/mL). Furthermore, in the close to ‘No-Kill’ group, the inhibition was either weaker or not significant. However, in both cases, the growth of the isolates themselves was significantly impaired at both 24 and 48 h (Figs. S1b-c).

**Figure 3.**
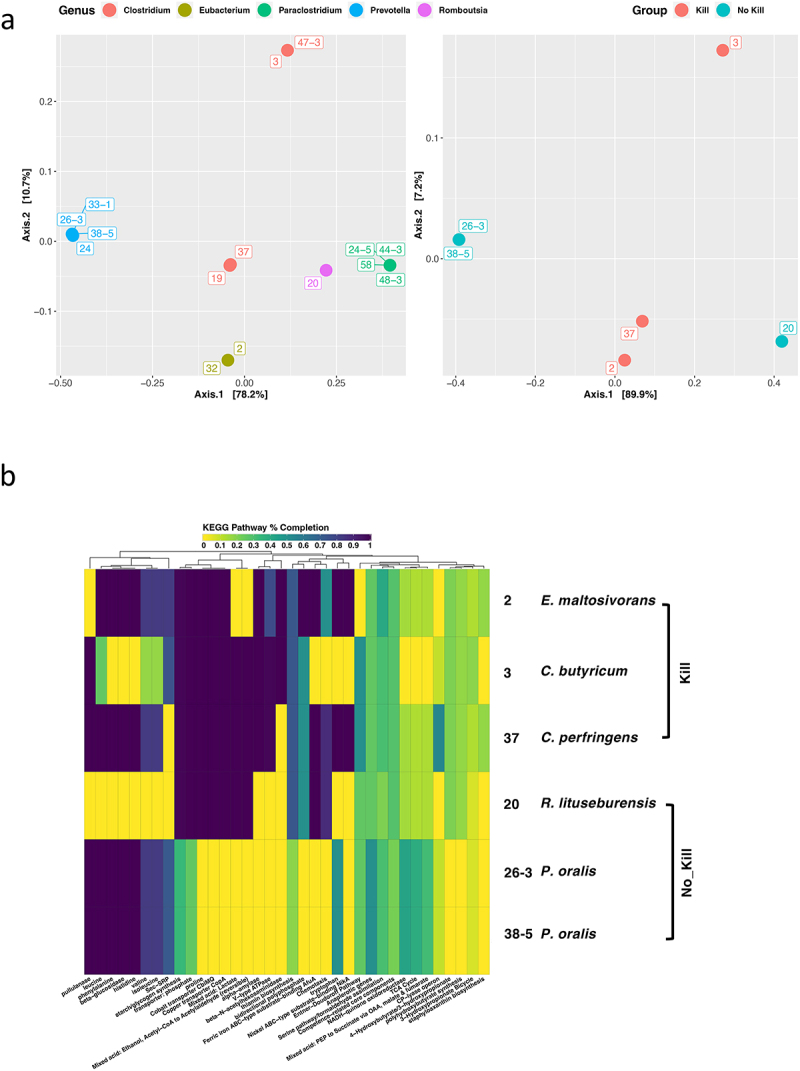
Evaluation of the functional genomic potentials among gut microbiota isolates.

We queried the 45,490 protein-coding genes predicted across the 15 gut microbiota isolate genomes using the Cluster of Orthologous Genes (COG) and Kyoto Encyclopedia of Genes and Genomes (KEGG) databases to survey the genetic underpinnings associated with their predicted metabolic capabilities.^34,35^ Principal coordinate analysis (PCoA) of functional profiles comprised of fractional abundances of genes with annotations to different COGs demonstrated a high degree of variation in functional potential among the subpopulation of ACHIM gut microbiota (Fig. S2a). This observed functional genomic diversity was underscored by results from similar analysis with KEGG pathway completion profiles generated for each of the 15 isolates (Figure 3a). PCoA analysis with COG fractional abundance and KEGG pathway completion profiles for isolates with established “Kill” and “No-Kill” *Salmonella* co-culture phenotypes revealed substantial variation in functional genomic potential between the two groups except isolate 20, which presented itself as an interloper with great distance from ‘No-Kill’ isolates 26–3 and 38–5 (Figure 3a and Fig. S2a).

(a) Principal coordinate analysis (PCoA) of KEGG pathway completion profiles for the 15 isolates selected for downstream functional analyses (left) and six isolates assigned to the “Kill” and “No-Kill” groups based on results from the *Salmonella* co-culture assays (right). The KEGG pathway completion profile for each isolate consisted of the 36 pathways surviving conditional filtering in R. Colors indicate the WGS-based taxonomic classification of each isolate at the genus level or the phenotypic group. (b) Hierarchical clustering of KEGG pathway completion profiles for the six isolates assigned to the “Kill” and “No-Kill” groups based on results from the *Salmonella* co-culture assays. Heatmap color indicates percent completion for each of the 36 selected metabolic pathways, expressed as a fraction (0 – 1). Darker colors correspond to higher metabolic pathway completion with lighter colors corresponding to less complete pathways.

Constructing COG functional category and KEGG Pathway completion annotation summaries for the “Kill” and “No-Kill” isolates and observing profile similarities among them provided additional insight into differences in functional genomic potential across these two phenotypic groups (Figure 3b and Fig. S2b). Both methods demonstrated two *Prevotella* isolates (26–3 and 38–5) with near-identical functional genomic capabilities, while the remaining four isolates exhibited numerous differences in COG abundance and KEGG pathway completion. The *E. maltosivorans* 2 genome exhibited higher frequencies of COG-category annotations in energy production and conversion such as V-type ATPase, coenzyme transport and metabolism including alpha-amylase, and K (transcription) relative to the other isolates (Figure 3b and Fig. S2b). In addition, *E. maltosivorans* displayed the highest degree of KEGG pathway completion among all six of the isolates investigated. However, unlike *E. maltosivorans* 2, the *Clostridium* members of the “Kill” group exhibited the highest COG frequencies for carbohydrate transport and metabolism (Figure 3b and Fig. S2b). Although the KEGG and COG profiles for both *Clostridium* isolates in the ‘Kill’ group showed high degrees of overlap, the *R. lituseburensis* 20 from the ‘Not-Kill’ group had quite a few similarities in KEGG pathways completion profile elements to those of the ‘Kill’ group isolates (Figure 3b and Fig. S2b).

### Metabolites unique in co-culture systems inhibiting *Salmonella* growth

**Figure 4.**
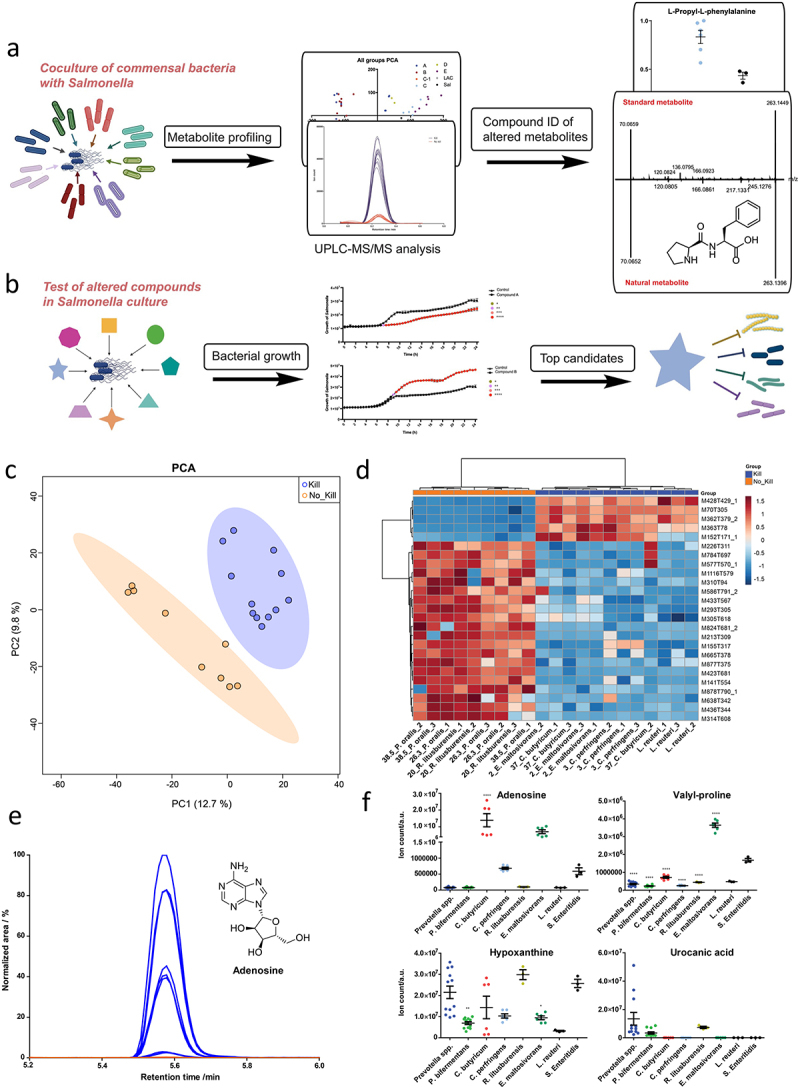
Metabolites unique in co-culture systems inhibiting *Salmonella* growth.

To discover the bioactive metabolites that cause the inhibition at the molecular level, we performed a mass spectrometric metabolomic analysis to profile metabolites after coculturing these selected 15 gut microbial strains with *Salmonella* (Figures. 4a-b). A monoculture of *Salmonella* and a YCFA culture were used as reference and control, respectively. The analysis was performed for each culture with biological triplicates. Principal component analysis (PCA) and heatmap clustering (top 25 mass spectrometric features based on one-way ANOVA) of the different bacterial strains revealed a clear difference between the species *P. phocaeensis* (A), *P. bifermentans* (B), and *Rombustia* (D) in comparison to the species *Clostriduium sp*. (C1), *C. perfringens* (C), and *E. maltosivorans* (E) (Fig. S2c). To identify metabolites that influence the growth of *Salmonella*, further targeted comparison was performed with the species that impaired *Salmonella* growth (*E. maltosivorans* 2, *C. perfringens* 3, *C. butyricum* 37, and *L. reuteri*) and bacteria that did not alter *Salmonella* growth (*R. litusburensis* 20, *P. oralis* 26–3, and *P. oralis* 38–5) (Figure 4c). The metabolite structures were validated through collision-induced fragmentation (CID). This structure identification process was performed for 214 features in the positive mode and 152 features in the negative mode mass spectrometry analysis (Table S3). Applied criteria were i) *p*-value <1.0 × 10^−5^ via t-test analysis; and ii) fold change > 10 to specifically focus on the highly altered metabolites. All obtained fragmentation spectra were compared with either experimental or computational mass spectrometry fragmentation libraries as well as our in-house library. Fragmentation spectra that did not match any of the previously available databases were uploaded into the computational tool SIRIUS for identification of the fragmentation fingerprint. The putative spectra were then either validated with chemical standards available in our in-house library or purchased. The final step of the metabolite structure validation was the comparison of the retention time of reference compounds with the natural metabolites in the bacterial cultures. After separate determination of the retention times, the chemical standard is utilized to verify the retention time in the biological matrix. Compounds with identical MS/MS fragmentation patterns and retention times were classified as confidence level 1 (Fig. S2d). In total, 100 molecules were identified and validated at the highest confidence levels with either authentic metabolite standards through co-injection experiments or comparison with databases for MS/MS fragmentation spectra.

(a-b) Schematic representation of the metabolomic analysis of the co-culture system and the following functional assays. The metabolite profile of the co-culture system of gut commensal isolates and *Salmonella* by an ultra-performance liquid chromatography-tandem mass spectrometry (UPLC-MS/MS) method was analyzed to identify the up- and down-regulated compounds in the co-culture system compared to the *Salmonella* control group. The impact of the altered compounds on *Salmonella* growth inhibition by culturing *Salmonella* with these compounds for 24 h was tested. Finally, the effect of the top candidates on other pathogens including multidrug-resistant pathogens was investigated. (c) Principal component analysis of the “Kill” vs. “No Kill” groups. (d) Heatmap of top 25 features (ranked by *p*-value) from the comparison between the “Kill” and “No Kill” groups. Darker red indicates higher concentration of the metabolites and darker blue indicates lower concentration of the metabolites. (e) Representative extracted ion chromatogram (EIC) of the adenosine from the “Kill” (blue lines) vs. “No Kill” groups (red lines). (f) Variations of mass spectrometric intensities between bacterial groups of the four validated metabolites adenosine, valyl-proline, hypoxanthine, and urocanic acid. Data were analyzed by one-way ANOVA with Tukey’s multiple comparison test. The significance indicated above each group is from the comparison with the *Salmonella* control group. *p < .05, **p < .01, ***p < .001 and ****p < .0001.

To illustrate our results, the top 25 mass spectrometric features in positive mode analysis are depicted in Figure 4d. We anticipated identifying the most affected metabolic differences between the two groups, as exemplified by adenosine (Figure 4e). The isolates of the same species demonstrate a highly similar metabolite pattern. To obtain metabolites structures at the highest confidence level, compounds of interest were identified with authentic synthetic standards including adenosine, adenine, agmatine, phenylalanine, glutamine, guanine, and the dipeptides Leu-Pro, Val-Pro, Leu-Thr, Pro-Phe, Try-Pro, and Tyr-Pro. Dipeptides are not a common compound class in metabolomic analysis and it is important to identify the correct order/sequence of the dipeptide for the follow-up bioactivity assays. We have determined the order of the two amino acids based on their specific fragmentation as previously reported by us.^36^ These molecules were present at significantly higher levels in at least one of the *Salmonella*-depleted co-cultures compared to the co-cultures with unaltered *Salmonella* (Figures 4f, 6a and Fig. S3, and Table S4 and Table S5). We have also included dipeptides in this list that were found to be significantly increased in the *L. reuteri* culture as these may also represent bioactive compounds. The levels of hypoxanthine, urocanic acid, and xanthurenic acid were found to be reduced in the co-cultures with reduced *Salmonella* growth (Fig. S3). Hypoxanthine is an intermediate of the purine degradation pathway, which is utilized by the microbiome as a substrate and is thus known as an important metabolite for metabolic microbial interaction.^37,38^ Urocanic acid is a metabolite of histidine degradation, which has been suggested as a potential signaling molecule and a cue for bacterial pathogenesis.^39^ Xanthurenic acid is a quinoline carboxylic acid and part of the tyrosine catabolism pathway. Minor antimicrobial activity has been reported for this compound for *E. coli* and *B. subtilis*.^40^

### Adenosine and adenine potently inhibit multiple antibiotic resistance pathogens

**Figure 5.**
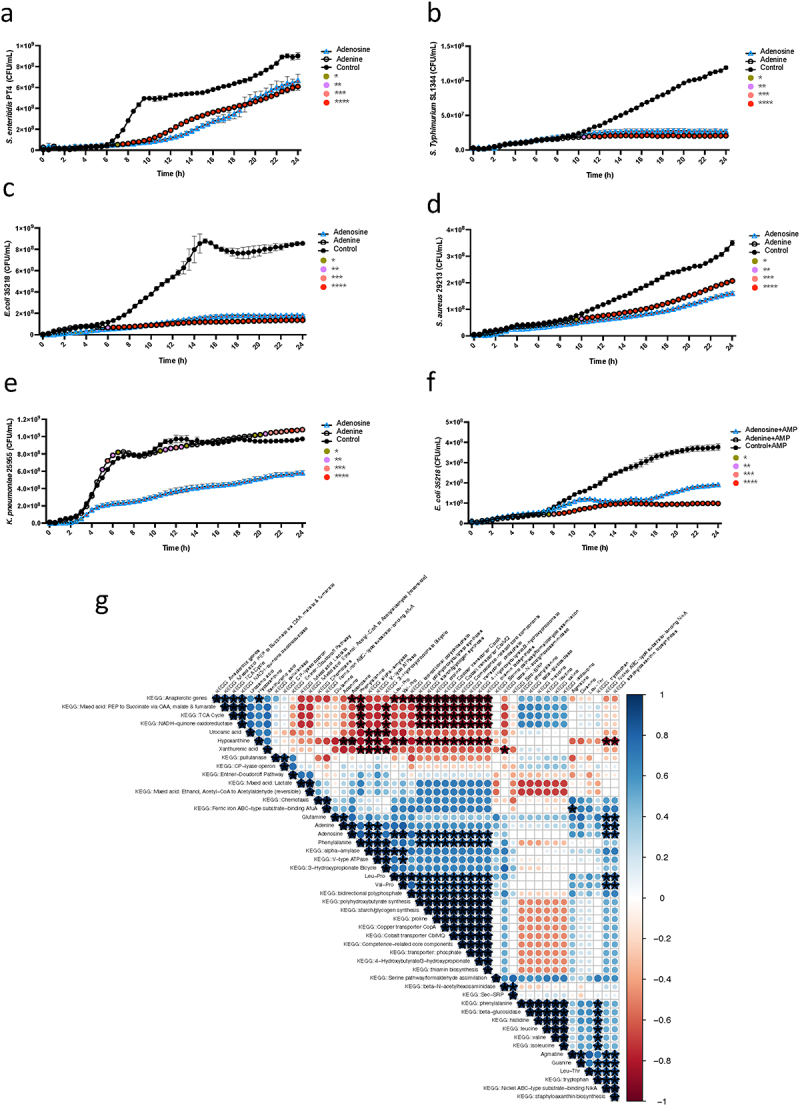
Adenosine and adenine are potent inhibitors of multiple-antibiotic-resistant pathogens.

After testing dose-dependent inhibition for adenosine and phenylalanine, 1 mmol/L was chosen to validate the findings on significantly changed metabolites (Fig. S4a-b). *Salmonella* was cultured with the top metabolites that were significantly changed (both increased and decreased) in the co-culture systems when comparing the *Salmonella*-depleted co-cultures and those without *Salmonella* inhibition. The growth of bacteria showed that among the tested compounds, adenosine and adenine demonstrated strong inhibition of *Salmonella* growth after 8 hours of culture (adenosine *vs*. control: 6.32 × 10^7^ CFU/mL to 2.83 × 10^8^ CFU/mL; adenine *vs*. control: 6.40 × 10^7^ × 10^5^ CFU/mL to 6.63 × 10^8^ CFU/mL) (Figure 5a). Other metabolites were increased in the *Salmonella*-depleted co-cultures including agmatine, phenylalanine, glutamine, guanine, and decreased in the *Salmonella*-depleted co-cultures, including hypoxanthine, urocanic acid, and xanthurenic acid did not significantly affect *Salmonella* growth (Fig. S4c-i).

(a) The growth of *Salmonella* co-cultured with adenosine or adenine for 24 h was compared with its growth in culture medium without adenosine. Data was analyzed by two-way repeated measures (RM) ANOVA with post hoc test Tukey’s multiple comparisons test. (b-e) The effects of the adenosine or adenine on the growth of listed pathogens for 24 h were compared with the growth of the pathogens in culture media without adenosine or adenine. (f) The effects of adenosine or adenine in combination with ampicillin on the growth of *E. coli* 25218. The CFUs were inferred from the OD values (OD = 1 equals *Salmonella enteritidis* PT4: 5 × 10^8^ CFU, *Salmonella Typhimurium*: 1.7 × 10^8^ CFU, *Staphylococcus aureus*: 1.7 × 10^8^ CFU, *E. Coli* ST131: 2.1 × 10^8^ CFU, *E. coli* 35218: 2.8 × 10^8^ CFU, *Klebsiella pneumoniae* 25955: 3.7 × 10^8^ CFU). Three independent assays were performed, and the data from the mean ± SD of triplicates from one presentative experiment was shown. *p < .05, **p < .01, ***p < .001, ****p < .0001. (g) Correlogram visualization of Spearman Rank correlation analysis of 36 KEGG pathways surviving conditional filtering and metabolite abundance values and the 12 compounds presented in Figure 4f and Fig. S3. Each correlation calculation included values from the six isolates assigned to the “Kill” and “No-Kill” groups based on results from the *Salmonella* co-culture assays. Significant correlations are marked with an asterisk (*p* < .05). Plot labels with the prefix, ‘KEGG:’ distinguish KEGG metabolic pathway completion variables from those that represent metabolic compound abundances. Metabolite abundance values represent the average abundance over triplicate sampling. Larger circles show higher correlation coefficients, with blue corresponding to positive correlations and red to negative correlations.

To further examine the potential inhibition of adenosine and adenine, we co-cultured adenosine and adenine with several other common pathogens including antibiotic-sensitive strain *K. pneumoniae* 25955, and multiple drug-resistant pathogens *S. Typhimurium* SL1344 (resistance to Streptomycin and Rifampin), *S. aureus* ATCC 29213 (resistance to Ciprofloxacin, Clindamycin, Gentamicin, and Neomycin), *E. coli* ST131 (resistance to Ampicillin, Cefotaxime, Ciprofloxacin, nalidixic acid, and Cefpodoxime), and *E. coli* 35218 (resistance to Ampicillin and Chloramphenicol).^41,42,43,44,45^ The growth of bacteria revealed that both adenosine and adenine completely inhibit the growth of *S. Typhimurium* SL1344 and *E. coli* 35218 in 24 h of co-culture (Figure 5b-c). Notably, the inhibition is strain-specific, as both adenosine and adenine did influence the growth of *E. coli* ST131. However, the inhibition effect on *E. coli* ST131 disappeared after 13 h (Fig. S4j). The growth of *S. aureus* was reduced but not completely inhibited by either adenosine or adenine, while only adenosine led to partial inhibition of *K. pneumoniae* growth (Figure 5d-e). Further investigation of the synergistic potential of adenosine or adenine with a minimum inhibitory concentration (MIC) breakpoint level for ampicillin (32 µg/mL) on *E. coli* 35218 and *E. coli* ST131 was further tested (Figure 5f and S4k). However, the addition of adenosine or adenine together with ampicillin did not increase the susceptivity of the pathogens *E. coli* 35218 and *E. coli* ST131. A similar concentration of adenosine and adenine did not significantly inhibit the representative gut microbiome isolates (Fig. S4l-m).

The correlation analysis between the top altered compounds and the KEGG pathways identified through WGS analysis revealed potential connections with the inhibition function. For example, both Adenine and Adenosine are associated with alpha-amylase, V-type ATPase, Nickel ABC-type substrate-binding NikA, and staphyloaxanthin biosynthesis pathways. Additionally, Adenosine is specifically linked to 4-Hydroxybutyrate/3-hydroxypropionate, thiamin biosynthesis, transporter: phosphate, Cobalt transporter CbiMQ, Copper transporter CopA, proline, and starch/glycogen synthesis (Figure 5g and Table S6).

### Opposing roles of dipeptides on *Salmonella* growth

**Figure 6.**
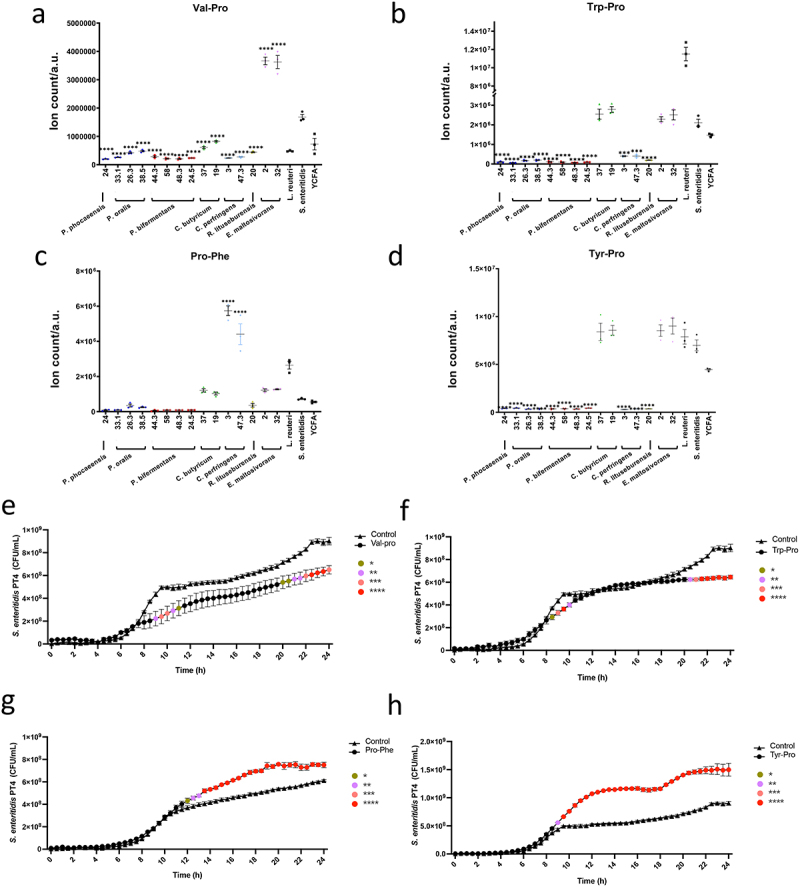
Dipeptides demonstrated the opposite function in *Salmonella* growth.

Among the top metabolite candidates, we identified several dipeptides including Val-Pro, Trp-Pro, Pro-Phe, Tyr-Pro, Leu-Pro, and Leu-Thr that were significantly higher in the supernatant from the *Salmonella*-depleted co-cultures or the *L. reuteri* co-culture (positive control) compared to the co-cultures with unaltered *Salmonella* (Figures 6a-d, Fig. S3j-k, Table S3, and Table S7). These dipeptides have not been well characterized before, especially on their potential function on infection. To investigate their potential roles, we supplemented *Salmonella* with synthetic dipeptides and monitored the *Salmonella* growth for 24 h. Interestingly, Val-Pro and Trp-Pro demonstrated inhibition of *Salmonella* growth after 24 h, while Pro-Phe and Tyr-Pro demonstrated the opposite function that boosted *Salmonella* growth starting at 8-h *Salmonella* (Figures 6e-h). Tyr-Pro showed the strongest growth promotion among all the tested compounds. No significant difference was found for related other dipeptides (Fig. S4n-o). These findings suggest that the identified dipeptides may have opposing functions in *Salmonella* infection, with some such as Val-Pro and Trp-Pro potentially secreted by commensal bacteria to combat *Salmonella*, and others such as Pro-Phe and Tyr-Pro produced by *Salmonella* as a defense mechanism. The study also highlights the complexity of the interplay between commensal bacteria and pathogens in the gut, as different dipeptides may have different effects on bacterial growth. Further investigation is needed to fully elucidate the functions of these dipeptides in infections and their broader implications for gut health.

(a-d) Level of the identified Val-Pro (a), Trp-Pro (b), Pro-Phe (c), and Tyr-Pro (d) by ultrahigh-performance liquid chromatography-tandem mass spectrometry (UPLC-MS/MS) in each co-culture supernatant of gut commensal isolates and *Salmonella*. Data were analyzed by one-way ANOVA with Tukey’s multiple comparison test. The significance indicated above each co-culture comes from the comparison with the *Salmonella* control group. *p < .05, **p < .01, ***p < .001, ****p < .0001. (e-h) The growth of *Salmonella* co-cultured with Val-Pro (e), Trp-Pro (f), Pro-Phe (g), and Tyr-Pro (h) for 24 h was compared with its growth in culture medium without dipeptide. The CFUs were inferred from OD value. Data were analyzed by two-way repeated measures (RM) ANOVA with post hoc test Tukey’s multiple comparison test. Three independent assays were performed, and the data from the mean ± SD of triplicates from one presentative experiment was shown. *p < .05, **p < .01, ***p < .001, ****p < .0001.

## Methods

### Bacterial strain isolations

The anaerobically cultivated human intestinal microbiota (ACHIM) culture is a cultivation of a healthy adult donor’s gut microbiota *in vitro*. ACHIM culture is stable at the species level and had been tested pathogen-free and applied in clinical trials.^10,21^ Our recent study observed that ACHIM significantly reduced *Salmonella* growth.^10^ To identify the specific active bacteria, single isolates were collected by sequential dilution of the ACHIM culture on *Deutsche Sammlung von Mikroorganismen und Zellkulturen* (DSMZ) modified Yeast Casitone Fatty Acids (YCFA) medium agar plates after 48 h of anaerobic incubation. Isolated gut commensal colonies were then anaerobically cultured in fresh modified YCFA liquid medium for these experiments.

*S. enteritidis* PT4 (*Salmonella*) was cultured in Luria-Bertani (LB) broth aerobically, and *Lactobacillus reuteri* DSM 17,938 was cultured in DeMan, Rogosa, and Sharpe (MRS) medium anaerobically before the commensal bacteria-*Salmonella* co- culture assay. The co-cultured of *Salmonella* with gut commensal isolates or *L. reuteri* as the positive control was carried out in DSMZ modified YCFA medium anaerobically. Bacteria for metabolite function assays (*S. Typhimurium* SL1344, *S. aureus* ATCC 29,213, *E. coli* ST131, *E. coli* ATCC 35,218, and *K. pneumoniae* ATCC 25,955) were cultured aerobically in M9 minimal medium (glucose 0.4%) with 1 mmol/L metabolite compound.

### Bacterial isolate WGS and bioinformatics analysis

The genomic DNA of each gut commensal bacterial isolate was extracted using the DNAeasy Blood & Tissue Kit (Qiagen, Germany) according to the standard protocols provided. DNA sequencing library preparation and DNA sequencing were performed following the protocols and standard pipelines for the Illumina MiSeq platform to generate 2 × 150 bp paired-end reads (Illumina, California, USA). FASTQ read pairs for each isolate WGS library were processed using the BACTpipe 2.6.0 workflow.^46^ Reads that passed quality assessment using BBDuk (http://sourceforge.net/projects/bbmap/) were subsequently screened for contaminants using Mash Screen.^47^ The surviving reads were then assembled into draft genomes using Shovill (https://github.com/tseemann/shovill). Pairwise comparisons of all isolate WGS assemblies for average nucleotide identity (ANI) were performed using the ‘sketch’ and ‘comparesketch’ scripts of the BBMap software package (http://sourceforge.net/projects/bbmap/). A genome similarity matrix based on pairwise ANI values was created by assessing the Pearson correlation coefficient for all ANI scores of unique isolate pairs using the ‘corr’ method of the Python module Pandas,^48^ followed by plotting with the ‘clustermap’ method from Seaborn.^49^

Taxonomic inference for each isolate based on WGS nucleotide composition was carried out using Sourmash 4.2.2.^50^ Briefly, MinHash sketches made for each isolate WGS assembly with a k-mer size of 31 and scaled setting of 2000 were queried against subjects in the genome taxonomy database (GTDB) r202.^33^ The ‘search’ sub-command was used to identify the ‘best-match’ between a subject in GTDB r202 (https://sourmash.readthedocs.io/en/latest/databases.html) and each ACHIM isolate WGS sketch. The ‘lca classify’ sub-command was used to generate taxonomic ranks for each of the 74 ACHIM isolates using the k-31 version of GTDB r202. The all-by-all pairwise comparisons of the Jaccard Similarity Index values between ACHIM isolate WGS MinHash sketches performed with the ‘compare’ subcommand were visualized in R 4.1 with the Complex Heatmap 2.10.0 package.^51^

Standardized bioinformatic pipelines for gene prediction using Prodigal 2.6.3.^52^ and corresponding functional annotation of protein-coding sequences (CDS) were implemented on ACHIM isolate WGS assemblies using automated workflows comprised within Bactopia 2.0.2.^53^ The multi-fasta amino acid sequence files generated by Prokka 1.14.6^54^ were used as inputs for eggnog-mapper v2,^34^ which was run with default settings using Diamond 2.0.11.^55^ Annotations from the Clusters of Orthologous Genes (COG) database^56^ were summarized into functional profiles for each isolate in R using functions from the Tidyverse 1.3.1 package.^57^ Adapted from previously reported methods, the fractional genomic abundances of each eggNOG orthologous group (OG) of genes^58^ present within isolate genomes were calculated in R and used to generate a sample-wise feature table.^59^ All COG functional profiles were filtered to keep only those present in all samples being analyzed for a given analysis. Functional distances based on eggNOG-OG abundances were determined by ordination with the R package Phyloseq using the PCoA method and Bray-Curtis dissimilarity.^60^

KEGG Pathway analysis of amino acid sequences generated by Prokka for each isolate was carried out using Kofamscan 1.3.0 with a default score threshold setting for KEGG Ortholog profile HMM assignment.^61^ KEGG-Decoder was used to assess KEGG pathway completion for each isolate.^62^ KEGG pathway completion results were filtered in R with 2 conditions based on the *Salmonella “*Kill” phenotype: (1) keep pathways with non-zero values in at least 2 out of 3 ‘Kill’ isolates and zero values in all ‘No_Kill’ isolates, and (2) keep pathways with zero values in at least 2 out of 3 ‘No_Kill’ isolates and non-zero values in at least 1 ‘Kill’ isolate. Correlation analysis between KEGG pathways and significant metabolite abundances was performed in R using the corrplot 0.92 package. Significant metabolite abundances used in correlation analyses consisted of mean values (*n* = 3). Correlation matrices and *p*-value estimation were carried out using the non-parametric Spearman rank correlation method. Significant correlations had estimated *p* ≤ 0.05.

### Commensal bacteria-*Salmonella* co-culture assay

Considering that the infectious dose of *Salmonella enteritidis* is approximately 1.5 × 10^4^ CFU/g, in our earlier publication, we employed 10^6^ CFU in a 10ml culture to ensure the establishment of infection, which could be monitored in our ACHIM system. To maintain consistency with our previous publications for comparison, a dosage of 10^6^ CFU was utilized in the current experiment.^10^ Each commensal gut isolate (1 optical density (OD) in 1 mL experiment volume) was co-cultured with 10^6^ CFU (1 × 10^6^ CFU/mL in 1 mL experiment volume) of *Salmonella* in culture tubes with modified YCFA liquid medium and incubated for 24 and 48 h anaerobically. *Salmonella* without commensal bacteria and *Salmonella* co-cultured with *L. reuteri* were used as controls.^63^ To quantify the bacteria growth, sequentially diluted co-culture was inoculated both on *Salmonella*-selective agar plates to get the *Salmonella* CFU and on YCFA plates to get the total bacteria number that could be cultured in the mix. The plates were cultured at 37°C for 48 h, aerobically for *Salmonella*-selective agar plates and anaerobically for YCFA plates as published.^10^ Bacteria numbers were counted from each condition and calculated by dilution factors. All experiments were repeated three times with triplicates. The co-culture assays performed with the isolates close to ‘Kill’ and ‘No_Kill’ followed the same steps and were repeated twice with duplicates.

### Metabolite extraction and ultraperformance liquid chromatography-tandem mass spectrometry (UPLC-MS/MS) analysis

Bacteria supernatants were filtered through a 0.2 µm filter before four times the volume of methanol (Fisher Scientific, USA) was added to precipitate large cell components and proteins. The solution was stored at −80°C until the metabolite extraction. An aliquot of the mixture (500 µL) was centrifuged at 13,500 rpm for 5 min (Eppendorf, USA) before the supernatant was dried under vacuum (SpeedVac – Eppendorf, USA) and re-dissolved in 100 µL of a 5% acetonitrile solution in water. Metabolites were analyzed by ultrahigh-performance liquid chromatography coupled with mass spectrometry (UPLC-MS) and identified through MS/MS fragmentation analysis, which differ among the three time points (Acquity UPLC system connected to a Synapt G2 Q-TOF mass spectrometer (Waters Corporation, USA)). The system was controlled through the MassLynx software package v 4.1 (Waters Corporation, USA). The metabolites were separated on an Acquity UPLC® HSS T3 column (1.8 µm, 100 × 2.1 mm) (Waters Corporation, USA). The mobile phase comprised 0.1% formic acid in MilliQ water (A) plus 0.1% formic acid in LC-MS grade methanol (B). The mobile phase gradient was set as follows: 0–2 min, 0% B; 2–15 min, 0–100% B; 15–18 min, 100% B; 18–20 min, 100–0% B; 20–25 min, 0% B, with the flow rate at 0.3 mL/min and column temperature at 40°C. The samples were transferred into the q-TOF via positive electrospray ionization. The capillary voltage was 2.50 kV and the cone voltage was set as 40 V. The source temperature was kept at 100°C, with the cone gas flow at 50 L/min and the desolvation gas flow at 600 L/h. The instrument was operated in MSE mode, with the scan range at m/z = 50–1200 and the scan time at 0.3 s. The instrument was calibrated using a solution of sodium formate (0.5 mM in 2-propanol: water, 90:10, v/v) and lock mass correction was done by using a solution of leucine-encephalin (2 ng/µL in acetonitrile: 0.1% formic acid in the water, 50:50, v/v) at an injection rate of 30 s.

### Functional assays of metabolite activity

To identify the effect of metabolite compound on pathogen growth, 1 × 10^6^ CFU/mL pathogen bacterial strain was cultured in 100 µL M9 minimal medium with 1 mmol/L metabolite compound in a 96-well plate format and incubated at 37°C for 24 h. The growth of bacteria was detected by the SpectraMax® i3× Multi-Mode Microplate Reader with absorbance at 600 nm (OD_600_) every 30 min. The pathogens cultured in 100 µL of M9 minimal medium without additional compounds was considered as control. The synergistic effects of the metabolites and the antibiotics were tested by co-culture *Salmonella* with the resisted antibiotics and metabolites in M9 medium for 24 h. To test the gut microbiome isolates, two commensal strains that exhibited significant inhibitory functions were cultured in 100 µL of YCFA medium under anaerobic conditions, as these strains did not grow under pathogenic conditions. A 1:100 dilution of overnight culture with OD = 1, supplemented with 1 mmol/L adenosine or adenine, was used as the starting concentration. Each experimental condition was repeated at least three times. Data were visualized by GraphPad Prism 9.

### Statistical data analyses

The difference in bacterial growth among different co-culture systems of ACHIM isolates and *Salmonella* were compared by the Kruskal–Wallis H test with post hoc tests and Dunn’s multiple-comparison test and visualized with GraphPad Prism software. The growth of *Salmonella* with metabolites in 24 h was compared to that of the control group with only *Salmonella* growing in culture medium by two-way ANOVA with post hoc test Šídák’s multiple comparisons test. The obtained UPLC-MS data comparing the different time points were analyzed using the XCMS software package under R (version 3.3.0) to perform peak detection, alignment, peak filling, and integration.^64^ Metaboanalyst was used for generation of the principal component analysis charts and heatmaps. Data were autoscaled before uni- and multivariate analysis.^65^

## Discussion

The discovery of bioactive metabolites produced by the commensal microbiome is a new and promising strategy with multiple opportunities to identify unknown antimicrobial compounds that inhibit pathogenic infection. This is of great importance, as the impending crisis of antibiotic-resistant bacteria that threatens millions of lives, especially those of pregnant women, infants, and people with weakened immune systems, and generates an enormous clinical and societal burden. Towards this goal, we have initially isolated over 100 commensal bacterial strains from a gut microbiota that demonsttated inhibition of *Salmonella* growth.^10^ We carried out whole-genome sequencing on all these bacteria and evaluated representative strains for their inhibition effect on *Salmonella* growth. The metabolomic analysis revealed that adenosine, adenine, agmatine, phenylalanine, glutamine, guanine, and several dipeptides, i.e., Leu-Pro, Val-Pro, Leu-Thr, Pro-Phe, Try-Pro, and Tyr-Pro, demonstrated significantly higher levels in these co-culture systems inhibiting *Salmonella* growth than bacteria that did not lead to any inhibition. Further validation with adenosine and adenine revealed direct inhibition of *Salmonella* growth. This observation was confirmed with multiple antibiotic-resistant pathogens. Overall, our study provides a powerful strategy for identifying and screening gut microbiome-derived metabolites against pathogens, especially multidrug-resistant bacterial strains.

All isolated strains from the *in vitro* commensal gut microbiota culture belong to the *Firmicutes* or *Bacteroidetes* phyla, which are from the two major phyla in the gut that represent about 90% of the gut microbiota.^66,67^ Many isolates from the *Firmicutes* phylum belong to the *Clostridium* genera, which usually represents 95% of the *Firmicutes* phylum.^66^ *P. phocaeensis* is the only species we isolated from the *Bacteroidetes* phylum. We further identified *C. perfringens*, *C. butyricum*, and *E. maltosivorans* but not *P. phocaeensis*, *P. oralis*, *P. bifermentans*, and *R. litusburensis*, isolates with strong inhibitory effects on *Salmonella* growth. This is in line with others showing that the spore-forming *Clostridia* species protected neonatal mice from *S. typhimurium* infection and a combination of *Enterobacteriaceae* and spore-forming bacteria from *Firmicutes* phylum conferred colonization resistance to *S. enteritidis*.^68,69^

Our metabolomics-based investigation of co-culture samples identified previously unknown bioactive microbiome-derived metabolites. Notably, similar to earlier publications, we identified that adenosine and adenine, both are components of purine metabolism, directly inhibit *Salmonella* growth.^70,71^ Nucleosides and their derivatives have been reported to show antibacterial activity by inhibiting cell-wall peptidoglycan biosynthesis and some bacterial enzymes, such as purine nucleoside phosphorylases and DNA ligases.^72,73^ Adenosine is highly abundant in the intestine and is estimated to be 5 mmol/L in the lumen of a normal human intestine and may reach 6 mmol/L in an inflamed intestine.^74,75^ Increased adenosine signaling suppresses excessive inflammatory reactions and protects against immune-mediated damage.^76^ Adenosine has also been reported to have a significant antibacterial effect against oral *Streptococci*.^77^ Adenosine analogues having cyclopentyl or deoxyribose groups in the N^6−^position exhibited good antimicrobial properties.^72^ Moreover, several adenosine analogues were reported to have antibacterial activity, such as *S. aureus*.^72,78^ Our correlation analysis between metabolites and WGS also demonstrated multiple potential pathways that these compounds could potentially affect. Interestingly, our data demonstrate that adenosine has a more broad-spectrum effect on several pathogens including multi-drug resistance strains *S. aureus* and several gram-negative bacteria: *S. enteritidis*, *S. Typhimurium, K. pneumoniae*, and *E. coli* 35218.

Adenine is a biosyntheitic precursor of adenosine.^79^ Adenine can also be formed by partial degradation of adenosine with de-glycosylation and has been reported to regulate the immunological functions of lymphocytes and restrain the inflammatory reactions induced by lipopolysaccharide (LPS).^80–83^ Multiple adenine derivatives have been reported to engage in antibacterial activity against both gram-positive and gram-negative bacteria.^84–86^ Our experiments support these findings and demonstrate a significant inhibitory effect of adenine on the growth of *S. aureus*, *S. enteritidis*, *S. Typhimurium*, and *E. coli 35,218*. However, adenine is not an ideal drug for infection clearance because a chronic low-dose adenine diet in rats has been widely used to establish chronic kidney disease models.^87,88^ Notably, both adenosine and adenine inhibited the growth of *E. coli* 35218 but not extended-spectrum *β*-lactamase (ESBL)-producing bacteria *E. coli* ST131. Moreover, synergistic effects have been reported for exogenous supplementation of metabolites that effectively improved antibiotic treatments against multidrug-resistant pathogens.^89,90^ Integrated biochemical screening and network modeling suggested that antibiotic-induced intracellular adenine limitation would increase ATP demand, leading to elevated central metabolism activity and enhanced antibiotic lethality.^91^ However, no synergistic effects of adenosine or adenine with ampicillin on ampicillin-resistant *E. coli* strains were observed in our study.

Dipeptides comprise a class of metabolites that have most commonly been described as downstream or degradation products of bigger proteins. Recently, two different studies have reported that the three dipeptides, i.e., Tyr-Leu, Phe-Leu, and Try-Leu, possess anxiolytic properties.^92,93^ The relevance of dipeptides has increased within the era of the microbiome, as several of these metabolites are produced or can influence microbiota or bacterial growth. Hsueh et al. have described the correlation between the levels of several dipeptides with interleukin-8 (IL-8) expression in the treatment of patients with bacterial pneumonia.^94^ Another publication details the effect of basic and bulky hydrophobic dipeptides in the acceleration of protein degradation through interaction with the ubiquitination sites.^95^ Additionally, the importance of regulating fecal microbiome was demonstrated in an increased level of the genus *Prevotella* in the human feces as both dipeptides promoted the growth of these bacteria.^96^ Most of the dipeptides have not been well studied although they are commonly found in the intestines. Herein, we identified and further investigated the six dipeptides (Val-Pro, Trp-Pro, Pro-Phe, Tyr-Pro, Leu-Pro, and Leu-Thr), and evaluated their impact on *Salmonella* growth. Our data demonstrates that Val-Pro and Trp-Pro exhibited weak yet significant inhibition on *Salmonella* after 24 h, while Pro-Phe, Tyr-Pro promoted the growth of *Salmonella*. These novel observations shed light on the potential mechanisms and new bioactive metabolites involved in the clearance of *Salmonella* infections and host defense mechanisms through unknown gut microbiome-pathogen interaction.

In conclusion, our study provides an interdisciplinary pipeline for the discovery of bioactive antimicrobial metabolites produced by the gut microbiome against pathogens. Strains from *C. perfringens*, *C. butyricum*, and *E. maltosivorans* were identified as the key microbes to inhibit *Salmonella* growth. Adenosine and adenine were discovered to directly inhibit the growth of pathogens including several multi-drug-resistant pathogens. We also demonstrated novel dipeptide features which suggest potential interactions and signaling between gut microbes and pathogens. We believe that our described powerful pipeline can be utilized for investigation of additional strains for discovery of metabolites with promising potential as new microbiome-derived drugs against antibiotic-resistant pathogens.

## Supplementary Material

Supplemental Material

## Data and materials availability

All the data generated from this study are included in this paper. Code used to analyze data and generate figures is available on GitHub (https://github.com/ctmrbio/achim_smh1). The WGS sequencing data for the isolates are available in the European Nucleotide Archive (ENA) database under accession project number PRJEB59984.
