# Supplementary figures and images for: The protective role of commensal gut microbes and their metabolites against bacterial pathogens
Source: Gut Microbes. 2024 May 26;16(1):2356275. doi: 10.1080/19490976.2024.2356275 (PMC11135852; doi:10.1080/19490976.2024.2356275)

Supplemental Figure 3

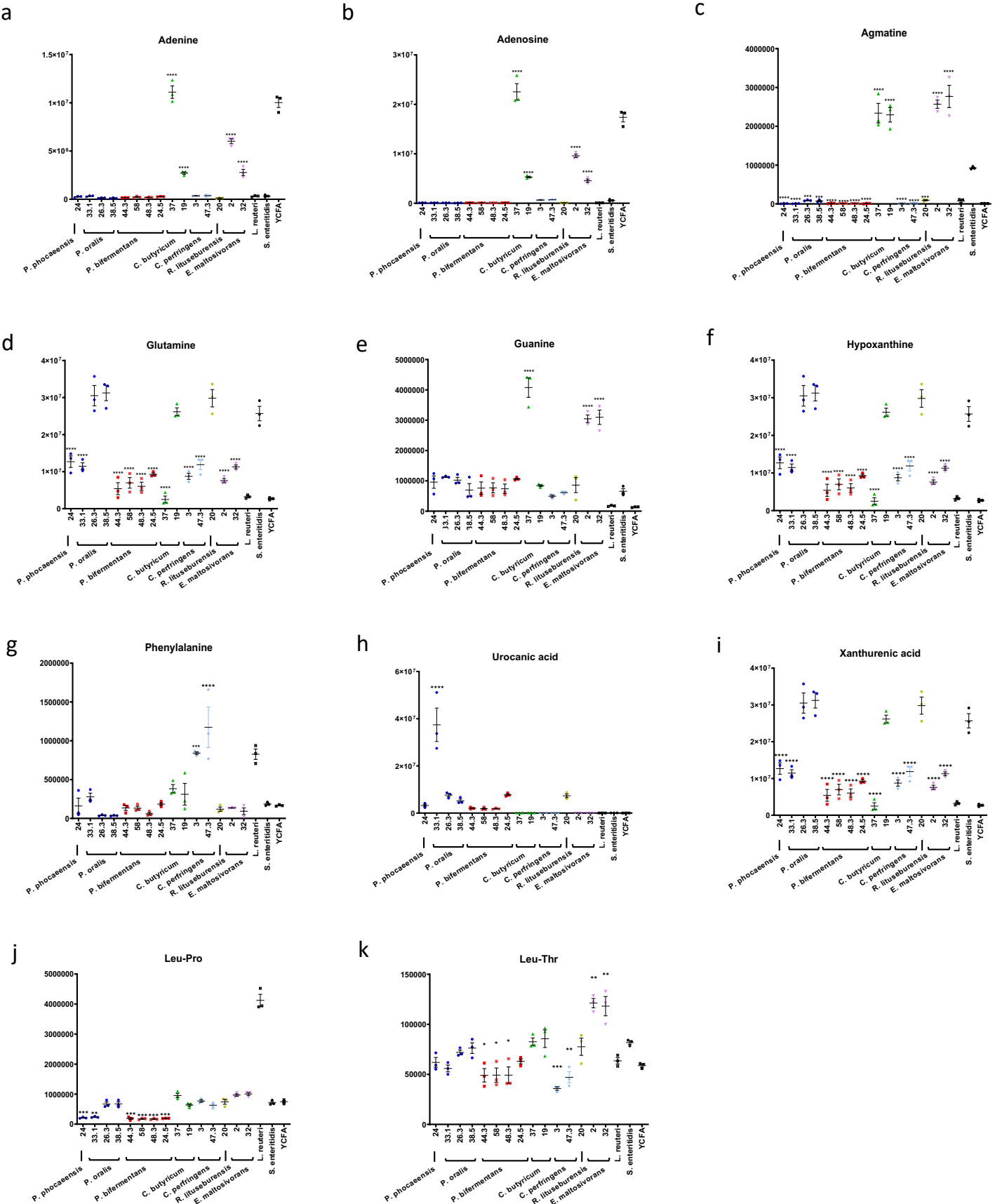

Supplement: Supplemental Material [file KGMI_A_2356275_SM6288.zip › Supplemental figures 3 proof reading.pdf]

Supplemental Figure 4

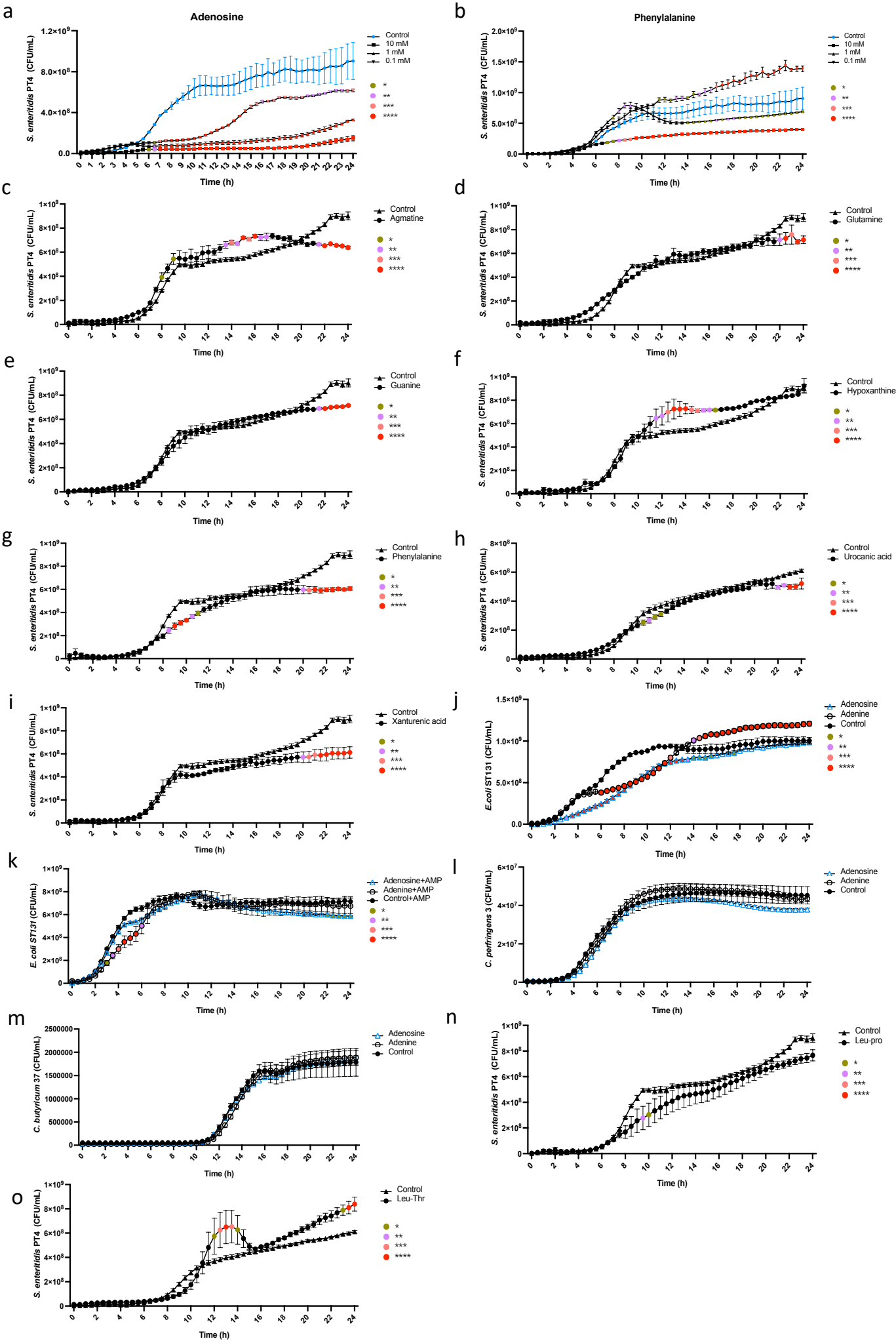

Supplement: Supplemental Material [file KGMI_A_2356275_SM6288.zip › Supplemental figures 4 proof reading.pdf]
